# Motion Capture Technologies for Ergonomics: A Systematic Literature Review

**DOI:** 10.3390/diagnostics13152593

**Published:** 2023-08-04

**Authors:** Sani Salisu, Nur Intan Raihana Ruhaiyem, Taiseer Abdalla Elfadil Eisa, Maged Nasser, Faisal Saeed, Hussain A. Younis

**Affiliations:** 1 School of Computer Sciences, Universiti Sains Malaysia, Gelugor 11800, Malaysia; hussain.younis@uobasrah.edu.iq; 2 Department of Information Technology, Federal University Dutse, Dutse 720101, Nigeria; 3 Department of Information Systems-Girls Section, King Khalid University, Mahayil 62529, Saudi Arabia; teisa@kku.edu.sa; 4 Computer & Information Sciences Department, Universiti Teknologi PETRONAS, Seri Iskandar 32610, Malaysia; maged.m.nasser@gmail.com; 5 DAAI Research Group, Department of Computing and Data Science, School of Computing and Digital Technology, Birmingham City University, Birmingham B4 7XG, UK; faisal.saeed@bcu.ac.uk; 6 College of Education for Women, University of Basrah, Basrah 61004, Iraq

**Keywords:** MBased systems, MLess systems, IMS systems, EMG, shoulder, hands

## Abstract

Muscular skeletal disorder is a difficult challenge faced by the working population. Motion capture (MoCap) is used for recording the movement of people for clinical, ergonomic and rehabilitation solutions. However, knowledge barriers about these MoCap systems have made them difficult to use for many people. Despite this, no state-of-the-art literature review on MoCap systems for human clinical, rehabilitation and ergonomic analysis has been conducted. A medical diagnosis using AI applies machine learning algorithms and motion capture technologies to analyze patient data, enhancing diagnostic accuracy, enabling early disease detection and facilitating personalized treatment plans. It revolutionizes healthcare by harnessing the power of data-driven insights for improved patient outcomes and efficient clinical decision-making. The current review aimed to investigate: (i) the most used MoCap systems for clinical use, ergonomics and rehabilitation, (ii) their application and (iii) the target population. We used preferred reporting items for systematic reviews and meta-analysis guidelines for the review. Google Scholar, PubMed, Scopus and Web of Science were used to search for relevant published articles. The articles obtained were scrutinized by reading the abstracts and titles to determine their inclusion eligibility. Accordingly, articles with insufficient or irrelevant information were excluded from the screening. The search included studies published between 2013 and 2023 (including additional criteria). A total of 40 articles were eligible for review. The selected articles were further categorized in terms of the types of MoCap used, their application and the domain of the experiments. This review will serve as a guide for researchers and organizational management.

## 1. Introduction

Human body motion tracking is currently one of the most expanding research areas. The term “motion capture” (MoCap) has been defined by different scholars depending on their respective research area. MoCap relates to the recording of the movement of objects or people. Various researchers [1,2,3,4,5] have identified two popular optical MoCap systems: marker-based (MBased) and marker-less (MLess) MoCap systems. Both systems have been used by many researchers to assess the ergonomic risks of industrial workers by capturing their body kinematics using smart cameras and transforming the information into three-dimensional (3D) data. However, researchers have extensively argued on which among the main MoCap systems is the best in terms of user satisfaction. Several studies have indicated that MBased MoCap is considerably accurate [6,7,8,9,10]. Other studies [5,11,12,13,14,15] have viewed that MLess MoCap is markedly appropriate. Among non-optical MoCap systems, inertial measurement unit (IMU) has been discussed as the best [16,17,18,19,20]. 

Medical diagnosis plays a crucial role in the field of healthcare, as it involves identifying and determining the nature of diseases or conditions in patients. Traditionally, medical diagnosis heavily relied on the expertise and experience of healthcare professionals. However, with the advancements in technology and the emergence of artificial intelligence (AI), there has been a significant transformation in the way diagnoses are made. AI-based medical diagnosis utilizes machine learning algorithms to analyze vast amounts of patient data, including medical records, imaging scans and genetic information, to assist healthcare professionals in accurate and timely diagnoses. This introduction explores the applications, benefits and challenges of AI in medical diagnosis, highlighting its potential to improve patient outcomes and revolutionize healthcare practices.

A number of systematic literature reviews and surveys on MoCap systems have been published, e.g., marker-less motion capture systems as a training device in neurological rehabilitation [21], the accuracy of motion capture systems for sport applications [22] and motion capture technology in industrial applications [23]. All of these reviews only considered single MoCap systems for either small groups or specific applications. Hence, presenting a systematic literature review on all MoCap systems is highly needed. Consequently, the purpose of this study is to assist researchers, healthcare practitioners and industrial managers to identify suitable MoCap systems in various applications of their need. For this, the present literature review was conducted to investigate (i) the most used MoCap system on ergonomics, (ii) their application and (iii) the target population and most-used body segments using the preferred reporting items for systematic reviews and meta-analysis (PRISMA) approach.

This systematic literature review is presented to address the research questions which include:

RQ1 Which brand is the most frequently used device in the MBased systems category?
RQ2 What is the main advantage of the Microsoft Kinect MoCap system compared to other systems in the MLess system category?
RQ3 What are some notable features and advantages of Xsens, CaptivL7000, IGS-180 and other systems that fall in to the IMU category?

The article is organized as follows: Section 2 describes the related literature of MoCap systems and a brief note on ergonomics. Section 3 presents the method used for the systematic literature review. Section 4 describes the results obtained from the method adopted. Section 5 gives the details about MoCap systems and the answers for the research questions are presented. Section 6 is the target population. Section 7 discusses and interprets the findings of the selected papers in the review. In Section 8, conclusions are drawn.

## 2. Related Literature on Motion Capture Systems

Effort has been exerted by many researchers using MoCap techniques to obtain workers’ data in their working environment and use such data in applying ergonomic principles to worker guidelines to reduce the risk of musculoskeletal disorder and improve productivity.

Ref. [24] used the Vicon 14 MX optical MoCap system to assess the potential risk of developing knee musculoskeletal disorder caused by residential roofing and determined that an awkward posture during sloppy roofing may have a significant impact on developing this disorder. MoCap was also used to analyze the relationship among body loads, experience and working procedure [25]. The outcome suggested that experienced workers adopt working techniques that are different from those of less experienced workers. MLess MoCap was reported to be the most cost-effective, efficient and easy to use [26,27,28], and demonstrated promising outcomes in occupational safety [29] and gait analysis [30]. IMU has been used by many researchers to diagnose the biomechanical overload of manual material handling workers [31] and analyze the motion of a healthy human wrist joint [32]. MoCap systems are used in several applications, such as sports, range of motion (ROM), ergonomics, health care, entertainment and advertisements. 

### Ergonomics 

Ergonomics is the scientific study of the relationship between man and his working environments. Numerous researchers and professionals have defined the term based on their respective areas of focus but they eventually turn out to have the same meaning. Research has shown that occupational safety and health administration (OSHA) support is highly required to reinforce workers’ knowledge in ergonomics and safety practices [33]. The inherent danger in hazardous occupations (e.g., construction, manufacturing, transportation, warehousing, mining, quarrying and healthcare services) and emergency services (e.g., firefighters, law enforcement and the military) results in substantial risks of occupational injuries [21,33]. Fitri and Halim [34] explained that most prevalent ergonomic-related injuries are musculoskeletal in nature, specifically caused by repetition, overload and an awkward posture in carrying out work. The musculoskeletal system (MSS) comprises the bones of the skeleton, muscles, cartilage, tendons, ligaments, joints and other connective tissues that support and bind tissues and organs. The MSS is responsible for providing shape, support, stability and locomotion to the body. Work-related musculoskeletal disorder (WMSD) is a painful disorder that affects workers’ MSS. Ref. [35] indicated that WMSD is a condition that affects the MSS and leads to pain and disabilities. MoCap data are essential for applying ergonomic principles to the guidelines for workers to reduce the risk of musculoskeletal disorder and improve productivity. However, obtaining accurate data is difficult owing to the nature of the working environment, heavy equipment used by workers, wearing personal protective equipment (PPE) and the limitations of MoCap systems.

## 3. Materials and Methods

This review used four different databases (i.e., Scopus, Web of Science, Google Scholar and PubMed) to search for relevant published articles or research in the field of applications of MoCap systems in ergonomics, healthcare and rehabilitation. The search queries used include some keywords and their combination to search for the relevant published papers within the publication years from 2013 to 2023: MoCap systems, MoCap technology, upper limb, lower limb, spine, ergonomics, gait, movement, kinematics, diagnosis and measurement. Given that our aim was to conduct a comprehensive review of research papers that suit the requirement of our study, a slight difference in the search strategies was adopted knowing the differences in the search capabilities of the selected databases. Title and abstract searches were performed in PubMed and Scopus from the beginning, while full text search was adopted in Web of Science and Google Scholar.

The articles obtained were scrutinized by reading the abstracts and titles to determine their inclusion eligibility. Those with insufficient or irrelevant information were excluded from the screening.

The full text of the searched papers were examined separately to determine the relevant information to enable their inclusion or exclusion. Furthermore, most of the references cited in the selected articles or papers were identified and used to retrieve more relevant papers for the review. To create clean and standard documents (i.e., no noise, no duplicates) retrieved from the different databases or sources, the following additional selection and rejection criteria were adopted.

- Articles should be original or reviews, written in English, and published in English journals or conferences.
- Any relevant articles published or in press between January 2013 and December 2023.
- The main focus being on MoCap applications on the ergonomics of human activities.

## 4. Results

**Figure 1:**
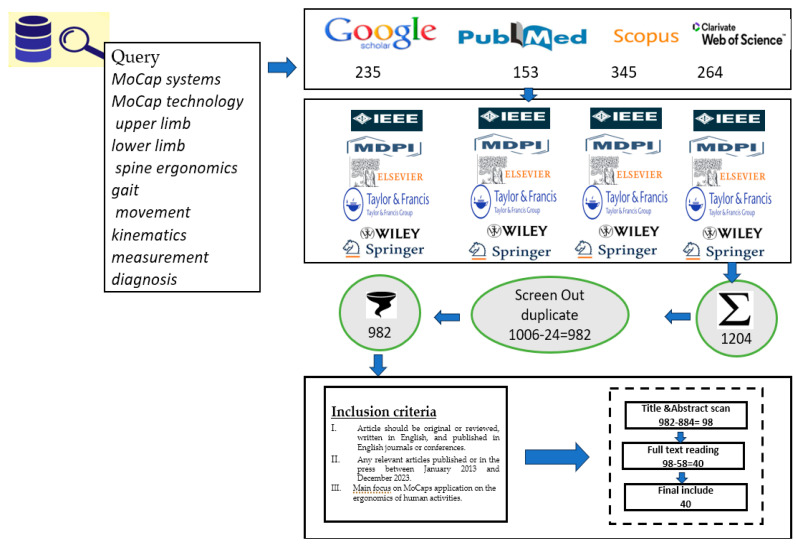
Selection of Studies, Search Query and Inclusion Criteria.

The computerized literature search resulted in 40 selected published studies on the application of MoCap systems in healthcare, rehabilitation and ergonomics, specifically discussing different human MoCap systems. A total of 1006 articles were first identified, with 24 duplicate articles discovered and excluded (*n* = 1006 − 24 = 982). A total of 98 articles were selected from (*n* = 982) after screening the title/abstract for further evaluation. Thereafter, 58 articles were further excluded following full-text reading, thereby resulting in the selection and analysis of only 40 relevant articles for the review. Figure 1 summarizes the stages of the article search and inclusion/exclusion process. The computerized literature search resulted in 40 published articles on the application of MoCap in ergonomics, healthcare and rehabilitation, particularly discussing different human MoCap systems. These systems are listed in this article. Given that most MoCap systems used in the selected literature are either MBase, MLess or IMU, these tables are titled MBased systems, MLess systems and IMU systems, respectively, with column titles as operational system, operational software, body segment used, number of body segment, measurement error and the domain of the experiments. The application of each MoCap system used is explained in the following section.

### 4.1. Distribution of Articles by Nationality of Authors

**Figure 2:**
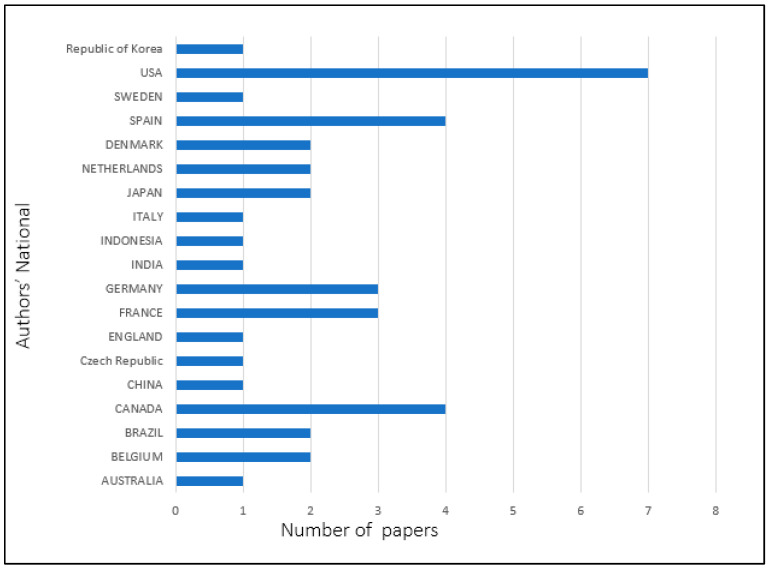
Distribution of selected papers by authors’ nationalities.

Figure 2 shows that 19 different countries used motion capture systems on healthcare, rehabilitation and ergonomic analysis in their studies. The selection was made by observing the countries where the studies were conducted. The distribution of 40 selected articles by the nationality of the authors shows that the United States of America has the highest number of published articles (seven), followed by Canada and Spain with four articles each. France and Germany published three articles each, while Brazil, Denmark, Japan, Belgium and Netherlands published two articles, respectively. Only one published article is found in Australia, Czech Republic, England, China, India, Indonesia, Italy, Republic of Korea and Sweden, respectively.

### 4.2. Distribution of Articles by Year of Publication

**Figure 3:**
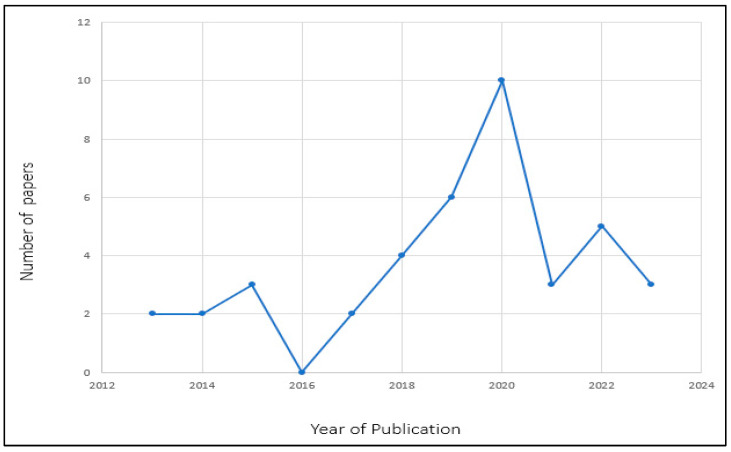
Distribution of selected papers by year of publication.

Figure 3 presents the distribution of articles by year of publication from 2013 to 2023, respectively. The number of included articles and their published year in the studies are described as follows. The year 2020 had the highest number of published articles (*n* = 10) which covers 25% of the total published articles in the study. This was followed by the year 2019 with six articles, covering 15% of the total published articles, followed by five articles published in the year 2022, covering 12.5%. Four articles were published in 2018 which covers 10% of the total published articles. Three articles were published in 2015, 2021 and 2023, respectively, covering 22.5% all together. Further, two articles were published in 2013, 2014 and 2017, respectively, which gives the total of 15% of all the published articles. No published article was found in the year 2016, hence 0% for the year 2016 is recorded.

### 4.3. Distribution of Article by Publishing Company 

**Figure 4:**
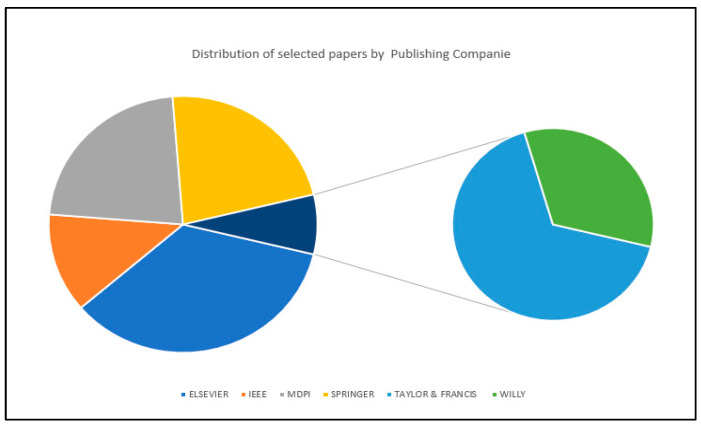
Distribution of selected papers by publishing companies.

Figure 4 shows the distribution of the selected articles used by their publishing companies. These selected articles are published by six different publishing companies which include IEEE (New York, NY, USA), MDPI (Basel, Switzerland), Elsevier (Amsterdam, The Netherlands), Springer (Berlin/Heidelberg, Germany), Taylor and Francis (Philadelphia, PA, USA) and Wiley (Hoboken, NJ, USA). The description of this distribution is as follows. From the figure, Elsevier published the highest number of articles used (*n* = 12), nine articles are published in both MDPI and Springer, five articles are published under IEEE, while two articles are published under Taylor and Francis and one article is published under Wiley.

## 5. Types of MoCap System

Different types of motion capture systems were used in the literature as shown in Table 1, Table 2 and Table 3. These motion capture systems are categorized into the MBased, MLess and IMU systems.

### 5.1. RQ1 Which Brand Is the Most Frequently Used Device in the MBased Systems Category?

For MBased systems, Vicon is the most frequently used device. Vicon MX3, MX13 and MX20 in the MX series and Vicon T-20 and T-40 are used in the T-series. Meanwhile, Vicon V16 and V5 are used in the V-series. MX represents the megapixels of the camera, such as MX-3+ (0.325 mega pixels). In the T-series, T-160 stands for 16-megapixel cameras, while the Vicon V family represents the vantage, indicating capture at high speed. A 3D MoCap system involves multiple high-definition cameras that are accurate, capable of capturing 370 frames per second at full frame resolution and can capture speeds of 2000 frames per second. Another MBased system used is CMOS. The system hardware was built using off-the-shelf components and the system can run at a rate of 63 frames per second. OptiTrack Flex3 is another system used in this category and consists of a small volume motion camera and is likewise affordable. This system uses six infrared cameras and spherical retroreflective markers of 14 mm diameter to output the marker information as XYZ data. Another MBased system used is OptoTrak. Eight OptoTrak motion tracking cameras were used to capture the 3D motion data of pelvis, hip and knee joints at 100 Hz. The system was used to validate the Kinect V2, used as the main system. The results of another study obtained from PhaseSpace were used to compare the results obtained by using the Kinect systems. Eight infrared PhaseSpace cameras were positioned around the capture space of approximately 4 m × 4 m. Moreover, the system provides the 3D position of LED markers with sub-millimeter accuracy and a frequency of up to 960 HZ. PhaseSpace enabled real-time data capture with under 10 ms latency. Table 1 summarizes the MBased systems.

**Table 1:** Mbased systems.

Study	Operational System	Operational Software	Body Segments	Number of Segment	Measurement Error	Domain of Experiment
[36]	Vicon T-40	MAS	Hand	1	MAE 5.75 mm	Ergonomics
[37]	Simple\camera	Kinematic inverse	Leg (Hip and Knee)	2	AVE 1.66 and 0.46	Ergonomics
[38]	CMOS and Kinect	Jack software	Whole body	Whole body	Nil	Ergonomics
[39]	ViconTH and iEMG	GraphPad StatMate 2.0	Upper Extremity (shoulder and elbow	2	Nil	Rehabilitation
[40]	PhaseSpace and Kinect1 and 2	PhaseSpace Recap2	Whole body	29 joints	76 mm and 87 mm	Ergonomics
[41]	Opti Track Flex3	Motive: Body	Upper body (hand and head)	2	Small	Ergonomics
[42]	Vicon (Oxford Metrics, Oxford, UK	ULMV 1.0	Upper Extremity	3	Nil	Rehabilitation
[43]	IR cameras, Xtion 3D sensor, and H4 Audio	Nexus 2.5	Head and hand	2	10 ms	Rehabilitation
[44]	Vicon MX13 and Xsens MTw	Nexus 2.0	Full body	all	Nil	Ergonomics

### 5.2. RQ2 What Is the Main Advantage of the Microsoft Kinect MoCap system Compared to Other Systems in the MLess System Category?

Under this category, Microsoft Kinect is the most frequently used MoCap system. It is an infrared MoCap device used for interactive computer games aimed for the Xbox 360 game console. Originally designed to replace the standard game controller, the device enables users to control and interact with the virtual reality environment through infrared cameras and depth sensors. This system can provide full-body 3D motion detection in real time. Microsoft Kinect is inexpensive, portable and easy to set up [45,46]. Move 4D is another MLess MoCap. Move 4D is a 3D human body motion scanner, modular photogrammetry-based 4D scanning system and consists of a set of 12 synchronized modules to scan full bodies with texture in motion. This system can capture up to 180 frames per second with a resolution of 2 mm. Table 2 presents the summary of MLess systems.

**Table 2:** MLess system.

Study	Operational System	Operational Software	Body Segment	Number of Segments	Measurement Error	Domain of Experiment
[47]	Microsoft Kinect	Microsoft SDK	Upper limb	4	Nil	Ergonomics
[12]	Microsoft Kinect and OpenSim	Open-Sim	Upper Extremity	4	Nil	Clinical
[48]	Kinect V2 and Vicon MX3	Nexus 2.5 and Microsoft SDK	Upper body	2	0.011 and 0.024	Ergonomics
[49]	Microsoft Kinect V2	Video annotation software, ELAN	Whole body	25 joints	Nil	Clinical
[50]	Kinect V2 and Optotrak	OpenTLD	Lower-Extremity	2	0.95 and 0.27	Clinical
[51]	Microsoft Kinect V2	OpesPose	Lower limb	1	Nil	Clinical
[5]	Microsoft Kinect V2, Captiv L7000	iPi soft	Upper-Extremity	2	<5.0	Ergonomics
[28]	Microsoft Kinect V2	Microsoft SDK	Lower limb	3	<5.0	Clinical
[52]	Move 4D	Move 4D	Whole body	1	Nil	Ergonomics

### 5.3. RQ3 What Are Some Notable Features and Advantages of Xsens, CaptivL7000, IGS-180 and Other Systems That Fall into the IMU Category?

Xsens was used more than any other system in this category. This system is a full-body MoCap system that integrates directly into the subject pipeline. It enables users to perform the capturing in all environments, as well as being known for easy calibration, real-time visualization, easy play back and capable of exporting and processing 3D data. CaptivL7000 is also an inertia system used under this category. This system is a flexible research software package for the synchronization of video and multiple measurements from TEA sensors and interfaced third-party hardware and measurement devices. IGS-180 is also used in this category. This system is Synergia’s professional level MoCap system, offering highly accurate and rich nuanced MoCap data. Moreover, this system is easy to use and does not need cameras capable of data capture at any given location, and there is no concern of occlusion or marker swapping. Thereafter, the MoCap system used is IMU, which uses accelerometers to capture more data on joint impact, limb movement and limb loads. In addition, this system is lightweight, easy to use, flexible and reliable. This system likewise enables field-based inertial measurements of impact and loads up to 200 g. It can capture the highest speed and highest impact sporting movements. An APDM Opal V2 inertial sensor is also used in one of the selected studies. Its sensors are placed on the subject body according to the manufacturer’s guidelines. Subjects were asked to walk on the GAITRite mat while wearing an APDM OpalV2 on each foot. Data were recorded simultaneously from the GAITRite and IMU systems [53]. Oqus300 is a MoCap device used in the experiment to capture seven retro-reflective markers that define the participants’ trunk segment. Another inertial system used is wireless sensor network (WSN). A human MoCap system based on inertial sensors and suitable for 3D reconstruction was designed to capture human posture data in the study. Ref. [54] added that “A WSN typically has little or no infrastructure. It consists of several sensor nodes (few tens to thousands) working together to monitor a region to obtain data about the environment”. The IMS systems are summarized in Table 3.

**Table 3:** IMU systems.

Study	Operational System	Operational Software	Body Segment	Number of Segments	Measurement Error	Domain of Experiment
[55]	IMUs	Mobile OS	Upper body	5	Nil	Ergonomic
[56]	Xsens MTw and Vicon V612	Xsens MTw	Lower limb	1	<1° and <3°	Clinical
[17]	IGS-180 and Vicon (MX20,	Nexus 1.8.2	Whole body	6	1.1°–5.1°	Clinical
[57]	Xsens MVN Link and Oqus 300 (IMC)	Xsens MVN studio 4.2.4	Lower body	1	>40%	Ergonomics
[58]	IMU and OMC	D-Flow	Lower limb	1	Nil	Rehabilitation
[59]	IMU (Xsens) and EMG	Xsens MTV studio pro.	Upper limb	4	Nil	Ergonomics
[60]	3IMUs and Vicon OXG	MTws Xsens	Lower limb	2	Nil	Clinical
[18]	IMUs and Vicon V5	Free IMU-GUI	Lower limb joints angles	1	0.63 and 1.2	Clinical
[61]	OptiTrack and EMG	OptiTrack	Upper-Extremity	2	Nil	Ergonomics
[62]	EMG and IMU	JMP software	Upper-Extremity	1	Nil	Ergonomics
[63]	APDM Opal V2	Moveo Explorer	Torso, Arms and Legs	3	Nil	Clinical
[64]	Wireless sensor network (WSN)	Truemotion	Whole body	1	Nil	Clinical

## 6. Target Population 

Different target populations with ergonomic problems were involved in the studies. The majority of the studies (*n* = 15) targeted the general population [17,18,28,39,40,44,50,52,58,60,64,65,66,67,68], twelve of which targeted the working population [5,38,47,48,55,59,61,62,69,70,71,72]. Six studies targeted the healthcare population [12,41,42,51,56,73], while other studies (*n* = 4) targeted sports persons [36,57,63,74]. Only one study [75] targeted university students. The remaining two studies targeted gesture and communication professionals [43,49]. Table 4 showcases the MoCaps system diagnosing different disorders from different populations.

**Table 4:** MoCaps System for Diagnostics.

Study	System	Sampling Frequency	Target Population	Sample Size	Diagnostic Outcomes
[39]	ViconTH and iEMG	200 HZ	General population	25	Lead clinicians to a more specific assessment and better intervention in upper extremity rehabilitation
[48]	Kinect V2 and Vicon MX3	Vicon100 HZ/Kinect30 HZ	Police, Traffic and Aircraft marshals	1	Kinect is an effective tool in tracking upper body motion
[38]	CMOS and Kinect	-	Assembly Operators In Aerospace Manufacturing	-	For fostering operation of an aircraft fuselage
[51]	Microsoft Kinect	30 HZ	Dementia Patients	14	The system can be used as a tool for monitoring of Parkinson’s in residential setting
[43]	IR cameras, Xtion 3D sensor and H4 Audio	100 HZ, 30 HZ and 44.1-KHZ	Deaf Translators	3	Used to investigate implicit detection of speech gesture
[41]	OptiTrak Flex 3	100 HZ	Surgeon	20	May improve skill acquisition and reduce physical stress during laparoscopic surgery
[36]	Vicon T-40	200 HZ	Swimmers	-	The system is accurate and feasible
[57]	Xsens MVT Link and Oqus 300	240 HZ and 120 HZ	Sportsmen	11	Using Inertia system, trunk speed is more accurate during walking than in transition period
[47]	Microsoft Kinect	30 HZ	Factory Operators	-	Kinect sensor is comparable to the Vicon system
[12]	Microsoft Kinect and OpenSim	-	Manual Wheelchair Users	-	The system is easy to use by clinicians
[65]	Vicon T20 and Vicon Bonita Video	-	General population	10	Allows a quantitative assessment of lower limb motion in the sagittal plane
[49]	Microsoft Kinect V2	-	Gesture and Communication professional	-	Can be useful to clinicians and researchers
[28]	Microsoft Kinect V2	30 HZ	General population	22	Kinect detects kinematic abnormalities of the trunk during slow walking on a flat land easier than on the treadmill
[42]	Vicon (Oxford Metrics, Oxford, UK)	100 HZ	People with Spinal muscular atrophy	17	Used for evaluating the need for clinical intervention
[5]	Microsoft Kinect V2 and Captiv L7000	30 HZ and 128 HZ	Manual operators in the industry	12	Kinect V2 accuracy reduced when occlusion occurs
[17]	IGS-180 and Vicon (MX20, T40)	60 HZ and 100 HZ	General population	20	The accuracy of joint kinematics can be affected when pairing a module unlike segment kinematics T
[18]	IMUs and ViconV5	128 HZ and 200 HZ	General population	7	IMU system is applicable in unconstrained rehabilitative contexts
[63]	APDM Opal V2	128 HZ	Female Gymnasts	8	The relationship between back pain and gymnastics training load/intensity is still not clear
[56]	Xsens MTw and Vicon V612	60 HZ and 120 HZ	Transfemoral amputees	1	The deviation of knee extension angle is found to be about 1
[50]	Kinect V2 and Optotrak	100 HZ and 100 HZ	General population		RGB data stream of Kinect sensor is efficient in estimating joint kinematics and unsuitable for measuring local dynamic stability
[40]	PhaseSpace (Impuls X2) and Kinect 1 and 2	480 HZ and 30 HZ	General population	10	Kinect 2 is more robust and accurate tracking of human pose as compared to Kinect 1
[55]	IMUs	100 HZ	Manual Workers in an Industry	12	The tool used can reduce the risk of musculoskeletal disorders in industrial settings
[64]	Wireless sensor network (WSN)	120 HZ	General population	240 sets of data	The system can meet the needs of doctors for real time monitoring of patients’ physiological parameters during clinical health monitoring
[44]	Vicov MX13 and Xsens MTw	100 HZ and 60 HZ	General population	12	Not suitable in real life situations
[61]	OptiTrack and EMG	120 HZ and 100 HZ	Firefighters and Emergency Medical Service	14	The system will reduce the biomechanical loads experienced by EMS providers when lifting and moving the patients
[52]	Move 4D	-	General population	-	The application is used for biomechanical analysis purposes
[62]	IMU and EMG	1500 HZ	Industrial Workers	14	Can be used to improve workplace design, injuries and enhance workers’ productivity
[58]	IMU and OMC	200 HZ	General population	3	Sensor network shows high accuracy in capturing significant gait parameters and features
[60]	3IMUs and Vicon OXG	100 HZ	General population	10	IMUs can be used to lower limb joint angle during straight walking
[59]	IMU (Xsens) and EMG	120 HZ and 2000 HZ	Banana production industrial workers	3	Bunches position, tools used by the workers and repetition movement led to musculoskeletal risk.
[66]	TTL-Pulse	200 HZ	General Population	15	Evaluating the performance of a motion capture device for diagnosing the risk of musculoskeletal disorder when doing physical activities
[75]	BR- BEWE TW		University students	425	Frequent risk of musculoskeletal disorder
[67]	Microsoft Kinect V2 and Vicon Bonita	100 HZ and 200 HZ	General Population	1	Potential health risks of the participants
[74]	QualisysAB,	100 HZ	Sport	16	To diagnose the kinematic differences among female Futsal players
[69]	MoCap suit—Axis Studio	90 HZ and 60 HZ	Operators working in automotive production	20	To predict the effect of bad working place on operators
[70]	IMUs	100 HZ	Workers form textile industry	93	To diagnose workers with lateral epicondylitis
[73]	Flexi 13, OptiTrack	100 HZ	Healthcare	10	Diagnosis and treatment of shoulder pain in rehabilitation homes
[71]	XSens MVN Link	240 HZ	Manual Workers	9	Diagnose the prevalence of work-related musculoskeletal disorders among the manual materials handlers.
[68]	STT-IWS, STT Systems and San Sebastian	100 HZ	General population	14	For effective diagnosis, assessment and treatment of spinal disorders
[72]	15 IMU	60 HZ	Workers on repetitive workstation circle	1	Compute the joint risks for every posture and output the total risk for the assessed workstation

## 7. Discussion

Choosing the right MoCap systems for ergonomic applications can be very difficult. Table 1, Table 2 and Table 3 may serve as a guide for researchers in making the right selection. Based on the result of this review, the majority of MoCap systems used in the selected articles were IMU-based (covering about 40%), while the camera-based systems (MBased and MLess) covered the remaining 60%, most likely due to the operational and processing cost and other technical challenges.

Outcomes revealed that the best selection of MoCap systems is mainly by the type of application. For example, quality control is achieved mainly via the use of the IMU system, while improving productivity via MBased and MLess systems. Another factor that warrants the use of MoCap systems is the environment; in uncontrolled environments, an IMU system is the best option, because the units can assess the performance of the subject throughout the experiment. However, in a controlled setting, e.g., laboratories, MBased and MLess systems will perform more accurately.

People’s wellbeing and safety was found to be the most common area of research in MoCap systems. For instance, all the studies in the selected articles focused on either ergonomic, clinical or rehabilitation research.

Other findings from this review revealed that when MoCap is combined with EMG, the musculoskeletal assessment of the subject was improved as well as the number of muscles to be analyzed; for example, biceps, triceps and forearm extensor strength muscle torques were measured with 0.2–2.000 as the measuring range [42] and EMG was used to investigate the physiological demand of right arm muscles involved in the bunch removal task [59]. It is obvious that neither MLess nor MoCap were combined with EMG in any the selected articles. Table 4 is showing the outcomes of the diagnosis of the subjects using the selected motion capture systems as presented above. AI-based medical diagnosis offers improved accuracy, efficiency and accessibility, but ethical and privacy concerns must be addressed. 

This review article is not perfect as it is attached with some limitations. There are many published articles relevant to MBase, MLess and IMU that may not be included in the review, to reserve future reproducibility. However, utilizing the PRISMA approach allowed us to identify a reasonable number of studies compared to some recent systematic literature reviews.

## 8. Conclusions

This systematic literature review has underscored how MoCap systems are utilized by researchers and organizational management to solve the issues of musculoskeletal disorder. The research was mainly driven by three experimental domains which include ergonomic, clinical and rehabilitation applications. In conclusion, the use of various technologies such as Kinect, IMU systems, sensor networks and motion capture devices has shown promising results in the field of medical diagnosis. These tools provide accurate and feasible assessments of various musculoskeletal parameters and can aid in diagnosing and monitoring conditions such as upper extremity rehabilitation, Parkinson’s, back pain, joint kinematics and work-related musculoskeletal disorders. However, challenges related to accuracy, occlusion, real-life applicability and privacy concerns need to be addressed for wider implementation. Overall, these technologies hold great potential in improving diagnosis, assessment and treatment in the field of medical diagnostics and workplace ergonomics.

The IMU system is the most-used MoCap system for such applications, as it relatively satisfies all the usability goals including the cost-effectiveness and displays minimal impact on the application domains of this research. Furthermore, the IMU system has long developed its performance in terms of low power utilization, logical partitioning and portability for easy body activity monitoring.

IMU systems may likely become the substitute of highly accurate but expensive MBased and MLess MoCap systems, especially with the current advancement that is making it smarter with built-in functions and embedded algorithms, such as deep learning and Kalman filters, that will process the data retrieved by IMU systems for more accuracy.

Moreover, systems need to be portable to interfere less with the subjects and workplace, while real-time assessments should go with health and safety applications to influence the acceptance and implementation of such technologies by researchers and organizational management.

MBased MoCap systems, such as vicon-T40 and PhaseSpace, come at a high cost and present high accuracy for some body activities and tracking tasks, but only in a controlled environment (e.g., laboratories). Attempts must be made to improve its usability. MLess MoCap systems, such as the Kinect series, are very low-cost compared to MBased MoCap systems, which also show high performance accuracy for specific classification and activity tracking tasks; nevertheless, efforts should be made to develop the tracking of more complex activities in real-time scenes. Finally, the ergonomic research domain has the highest number of articles in the selected publications.

## Data Availability

Not applicable.
